# Prostate Cancer Stem Cells: Research Advances

**DOI:** 10.3390/ijms161126036

**Published:** 2015-11-17

**Authors:** Dagmara Jaworska, Wojciech Król, Ewelina Szliszka

**Affiliations:** Department of Microbiology and Immunology, School of Medicine with the Division of Dentistry in Zabrze, Medical University of Silesia in Katowice, H. Jordana 19, 41-808 Zabrze, Poland; djaworska@sum.edu.pl (D.J.); wkrol@sum.edu.pl (W.K.)

**Keywords:** cancer stem cell, prostate cancer, metastasis, castration-resistant prostate cancer

## Abstract

Cancer stem cells have been defined as cells within a tumor that possesses the capacity to self-renew and to cause the heterogeneous lineages of cancer cells that comprise the tumor. Experimental evidence showed that these highly tumorigenic cells might be responsible for initiation and progression of cancer into invasive and metastatic disease. Eradicating prostate cancer stem cells, the root of the problem, has been considered as a promising target in prostate cancer treatment to improve the prognosis for patients with advanced stages of the disease.

## 1. Introduction

Prostate cancer continues to be the most common cancer diagnosed in male patients and the second leading malignancy of cancer-related deaths in Europe and the United States [1,2]. Most patients with advanced stages of the disease respond to the current treatment (hormonal therapy, radiotherapy, or chemotherapy) only in the beginning, mostly developing progression and subsequently widespread metastasis, resistant to prostate cancer therapeutic methods. Numerous basic research studies on the mechanisms of carcinogenesis did not significantly improve the prognosis and therapy outcome of patients with prostate cancer [3,4,5].

Early observations in the 1960s based on experiments conducted on blood neoplasms, proved cell heterogeneity within the tumor mass, suggesting the existence of immature cell population that may arise from the entire population of tumor cells [6,7]. The first evidence confirming the hypothesis of the existence of cancer stem cells derives from the 1990s and is based on the study of blood diseases such as acute myeloid leukemia (AML) [8]. The following years of research showed the presence of cancer stem cells also in other types of tumors, *inter alia*, prostate cancer [9,10,11].

Although still controversial, the cancer stem cell maybe the root cell of cancer and the most crucial target in the treatment of the disease. Therefore, the understanding of its biology might allow this cell type to be eliminated by targeted therapy, leading to improvement in therapeutic outcome [12].

Prostate cancer research is now focused on the cancer stem cells to get better understanding of the tumor initiation mechanisms, progression and metastasis formation, and it will eventually help to provide the patients with a better therapeutic effect [13,14,15,16].

This review is focused on the following aspects: the main differences between stem cells and cancer stem cells, prostate cancer stem cell identification and their molecular markers, current theories on the origin of prostate cancer stem cells, and finally the current research helping to understand biology of castration-resistant prostate cancer (CRPC) and develop new strategies to eliminate all cancer cells.

## 2. Stem Cells and Cancer Stem Cells (CSC)

Stem cells are immature cells characterized by significant proliferative properties, self-renewal, and the potential to differentiate into specialized cells in a tissue [17]. Stem cell division may be symmetric or asymmetric. A stem cell during asymmetric division gives rise to one mother cell that is identical to the cell of origin (has the same proliferative potential) and another daughter cell that will differentiate. Thus the population of stem cells remains constant. The other type of division results in two identical stem cells and it is defined as symmetric [18,19]. Fundamentally, there are two kinds of normal stem cells: embryonic which are pluripotent and can give rise to all cell types, and non-embryonic stem cells with a limited potential to differentiate into other cell types [17].

Cancer stem cells (CSCs) possess stem-like nature to a degree sufficient to compare them with normal stem cells. According to American Association for Cancer Research (a statement accepted in 2006) a cancer stem cell is “a cell within a tumor that possesses the capacity to self-renew and to cause the heterogeneous lineages of cancer cells that comprise the tumor” [20].

According to the definition, both normal and cancer stem cells share at least one feature—the ability to self-renew. The self-renewal is defined as the ability to produce posterity which retains stem-like characteristics. Therefore the division of cancer stem cell results in one or two daughter cells that have the same ability to proliferate and generate new cells—identical as the original, parental cell [19,20].

These cancer stem cells possess the capacity to extend the cancer stem cell population and moreover, after differentiation, become other cancer cell types which constitute the tumor bulk of cells [20,21]. The tumor is then organized in a cellular hierarchy, like it is arranged in the normal tissues. The non-stem cells in the tumor have high, but not unrestricted ability to proliferate. Therefore, the only cells within the tumor with unlimited potential are cancer stem cells, so they are capable of driving growth and metastasis [18,20]. Because the time between divisions in CSC is very long, it has been suggested that this phenomenon can be responsible for the resistance to treatment. For this reason, CSC which are relatively insensitive to therapies currently focused on eradication of dividing cells, may be a source of cancer relapse [22,23,24].

The main difference between CSCs and normal stem cells is their frequency of occurrence. Normal stem cells are present in the tissues in a very small amount. Contrariwise, CSCs can be a rather large population in the bulk of tumor cells [21]. The proportion of cancer stem cells in the whole tumor mass may be very different depending on the type of cancer, and it has been reported to have certain significance for predicting the prognosis [25,26,27].

CSCs are similar to normal stem cells in that they are long lived, slow cycling, self-renewing cells in undifferentiated state, which can generate a large number of differentiated progeny cells. CSCs frequently express many early developmental markers of normal stem cells. The difference lies in the regulation and control of their proliferation [20,28]. Normal stem cell functions are under strict control, while the divisions of cancer stem cells are out of control. There are also emerging evidences that cancer stem cells gain independence from factors suppressing their proliferation, including the role of their microenvironment (niche), and get the ability to occupy other niches [29,30,31]. These cells are programmed to maintain tumor growth and development by producing a large number of cancer cells [17,18,19,20].

## 3. The CSC Hypothesis

Two models of tumor development have been proposed; the stochastic model and the hierarchy model which assume the existence of CSCs. The stochastic model (which has only historical significance nowadays) predicts that any cell in the tumor has an identical probability to be the tumor-initiating cell, when it develops the ability to self-renew. This process is controlled stochastically. The second model, originally proposed in the mid-19th century by Rudolf Virchow is the model of cancer stem cells [32]. Although this concept is not new, because it has been discussed already decades ago, the identification and isolation of stem—like cells in human malignancies was not successful until 1997. This was accomplished by Bonnet and Dick in acute myeloid leukemia (AML) who showed that small subset of leukemic cells (CD34^+^CD38^−^) was able to initiate the disease when transplanted into a NOD/SCID mouse (non-obese diabetic/ severe combined immunodeficient mouse) [8]. Since then, this method has become the gold standard in stem cell research [20]. For the solid tumors proving the presence of cancer stem cells was much more difficult for several reasons. These cells are an integral part of the tissue which makes them less accessible. Furthermore the markers for cancer stem cells from solid tumors have not been investigated enough yet. Only several years later it was observed that CD44^+^CD24^−/low^ cells isolated from human breast cancer can induce breast cancer in NOD/SCID mice, while the remainder of cells failed to induce tumors. These innovative experiments suggested existence of cancer stem cells also in solid tumors [33]. In the last decade, CSCs have been identified and isolated in other solid tumors, including colon cancer [34], prostate cancer [35], hepatocellular carcinomas [36], or brain tumors [37] (Table 1). These studies provide evidence that tumor tissue is organized hierarchically, and only a sub-population of cells possesses the ability to initiate the tumor growth and survival.

**Table 1:** Cancer stem cell (CSC) in solid tumors and their first isolation year.

Cancer Types	Markers of CSCs	Year of Identification	Reference
Brain tumor	CD133^+^	2004	[37]
Breast cancer	CD44^+^/CD24^−^	2003	[33]
Colon cancer	CD133^+^	2007	[34]
Hepatic carcinoma	CD90^+^/CD45^−^/CD44^+^	2008	[38]
Lung cancer	CD133^+^	2005	[39]
Melanoma	ABCB5^+^	2008	[40]
Ovarian cancer	CD44^+^/CD117^+^	2008	[41]
Pancreatic cancer	CD44^+^/CD24^+^/ESA^+^	2007	[42]
Prostate cancer	CD44+/α_2_β_1_^(hi)^/CD133^+^	2005	[35]

Moreover, it was established for human AML stem cells which are capable of regenerating the tumor that these cells display CD34^+^CD38^−^ cell surface markers. This kind of phenotype is similar to one typical of normal human hematopoietic progenitors. It may suggest that AML stem cells arise from normal stem cells [20]. However this statement is not necessarily true for all types of cancer. Actually, three hypotheses for the origin of CSCs exist. One of them declares that CSCs come just from normal adult stem cells that have acquired many genetic mutations. Another hypothesis states that CSCs develop from tumor cells across cellular dedifferentiation through the EMT (epithelial-mesenchymal transition) pathway [43]. The third one is related to induced pluripotent stem cells (iPS) [44]. Nowadays, most studies support the hypothesis that stem cells existing in normal adult tissue are the targets of carcinogenesis and transformation. The accumulation of mutations is a long lasting process and can take even several years. In this context, the only cells that live long enough in adult organism are normal stem cells. This premise makes adult stem cells ideal candidates for the cells of origin for cancer stem cells. According to the theory of carcinogenesis, malignant transformation may occur as result of wide range of mutagenic agents acting on stem cells present in the adult tissue.

## 4. Prostate Cancer Stem Cells

### 4.1. Prostate Epithelial Stem Cells in the Adult Gland

**Figure 1:**
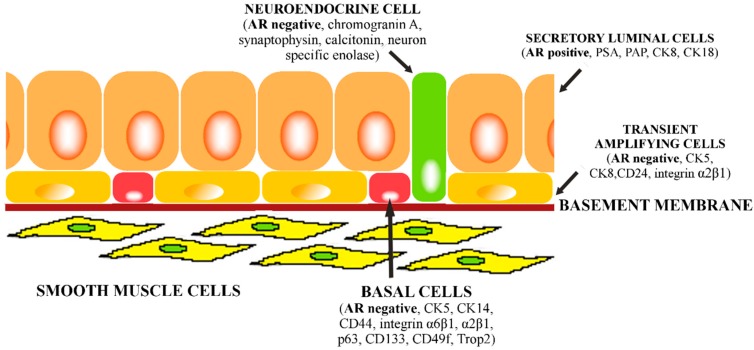
Schematic diagram of prostatic cellular compartments and their identity markers.

Normal, mature prostatic epithelium consists of three basic cell types: basal, luminal (secretory), and neuroendocrine that are identified by distinct marker expression. Additionally, there is a small group of intermediate cells that express both basal and luminal cell markers. These cells are referred to as transient amplifying cells (Figure 1).

The luminal cells are the most differentiated cells in the prostatic epithelium, express high levels of androgen receptor (AR) and low molecular weight keratins, secrete prostatic specific antigen (PSA), and prostatic acid phosphatase (PAP). Luminal cells are the main cell type in the epithelium and depend on androgens for survival in contrast to basal cells which do not express AR and are androgen-independent and undifferentiated [9,10].

Neuroendocrine cells are very rare cells located in the luminal epithelial layer. These differentiated cells express chromogranin A, synaptophysin, calcitonin, and neuron specific enolase (NSE) but do not express AR or PSA. Neuroendocrine cells are distributed in prostatic glands in all anatomic areas and they constitute less than 1% of prostatic epithelium [45,46].

The basal cells are located on the basement membrane in prostate gland epithelium. These cells are characterized by the expression of high-molecular weight cytokeratins 5 and 14 (CK5 and CK14) [47,48], CD44 [49], integrin α_6_β_1_ [50,51], and p63 (a member of p53 transcription factors family) [52].

Basal and luminal cells are hierarchically related. Basal cells are the progenitors of secretory cells, forasmuch as the existence of intermediate cells between these two types has been observed. These cells with an intermediate phenotype represent the transient amplifying cells [10,53,54,55,56]. One of the markers expressed on these cells is a CD24 surface molecule. CD24 distinguishes between low differentiated basal cells and transient amplifying cells and may play a significant role in the prostate gland cells differentiation [57].

A small number of cells among the population of basal cells have been recognized as the stem cells of the prostate both in mice and human. It is unclear how many subtypes of basal cells are present in the prostate and which subtype contains the main stem cell niche in the adult prostate [54,58].

Goldstein *et al.* [59] reported that specific marker, namely, tumor-associated calcium signal transducer 2 (TACSTD2/Trop2/M1S1/GA733-1) functionally discriminates between the two distinct basal cells subpopulations. Only the basal cells that express high levels of Trop 2 had the stem cell characteristics in the murine and human prostate [59]. Whereas, Lee *et al.* [58] identified seven basal cells subpopulations according to their p63, cytokeratin 14 and 5 expression. This group discovered that p63^+^/CK5^−^/CK14^−^ subpopulation contain self-renewable stem cells with the greatest potential for differentiation [58].

In the adult human prostate CD133 (also known as Prominin-1) expression is thought to be characteristic of stem-like populations based on their expression of α_2_β_1_ integrin and high clonogenic properties. Moreover, CD133 expression has been reported for prostate cancer stem cells [60,61]. CD133 expression is not restricted to the prostate gland; adult stem cells in other tissues also can often exhibit expression of this surface marker [62,63,64].

### 4.2. Origin of Prostate Cancer

The origin of prostate cancer remains controversial. The cell-of-origin of cancer—is the first cell which gains the mutations leading to cancer initiation. Whereas, cancer stem cells, defined by self-renewal and differentiation potential are the group of cells that maintain the tumor proliferation. The connection between both types is not completely understood yet. Their phenotypes may be different but they can also dynamically change. Two experimental approaches are used to characterize these two types of cells: transplantation assay and lineage-tracing assay [65]. Transplantation assay is a current “gold standard” for identifying cancer stem cells. This assay is based on xenografting isolated cells (with a specific phenotype) into immunodeficient mice. It has been used to prove the existence of cancer stem cells in several human cancers [20,33,34,35]. On the other hand, lineage-tracing assay is used to identify the potential cell-of-origin of cancer, however it can be also helpful in studying cancer stem cells. Lineage-tracing assay involves genetic labeling to determine individual cell fate. Then transformed, lineage-traced cells that formed a tumor can be analyzed to establish if they have cancer stem cells properties [65,66]. However, these classic types of experiments are not perfect in their design. Some authors impute that since they are performed in immune-deficient animals, they do not reflect the real state. If similar studies were done in immune-competent animals they would be more solid and reliable [67].

There are two possible cell-of-origin in prostate cancer, specifically—basal cell or luminal cell of origin. The prostate cancer cells usually have phenotype of the luminal cells, but they are not terminally differentiated as normal luminal cells. The cancer cells possess the unlimited proliferative capacity, unlike normal luminal cells, and they resemble more the basal cell characteristics. Firstly, it was assumed that the luminal cells were the source of all tumorigenicity, forasmuch as they constitute the bulk of the tumor mass. Nevertheless, several studies have brought evidence that prostate cancer stem cells are involved in the process of oncogenesis in the prostate gland. Basal cells in the prostate gland express surface molecules that regulate stem cell self-renewal such as p63, CD44, CD49f, CD133, therefore the prostate basal cells have been proposed to contain stem cells [7,35,68,69,70].

Cancer stem cells can arise from normal stem cells which are located in the basal layer of prostate gland. In the normal state, the stem cells can give rise to a second population—transient amplifying cells which subsequently differentiate into mature secretory cells [58,71,72]. It has been proposed that during carcinogenesis the normal stem cells accumulate mutations and are converted to highly tumorigenic and metastasis—initiating cancer stem cells. The main assumption was that cancer may arise as a result of genetic mutation in these cells, and this mutation concerns mainly oncogenes and tumor suppressor genes, in consequence resulting in uncontrolled cell growth [73,74,75,76,77,78]. It has been revealed that the tumorigenic prostate cancer stem cells can express specific markers such as telomerase, CD44, CD133, α_2_β_1_-integrin, multidrug resistance proteins, aldehyde dehydrogenase, and low or undetectable levels of AR. Moreover, several studies in prostate regenerative systems and xenograft mouse models confirmed that prostate cancer stem cells could play critical role in carcinogenesis, metastasis, and resistance to currently used therapies [73,75,76,77,79,80,81].

However, there is some evidence that supports an existence of luminal cells with stem activity. Several groups have investigated if luminal progenitor cells are luminal-restricted or not. It has been described earlier, that PTEN protein is involved in the stem cell self-renewal [82,83]. Korsten *et al.* [84] demonstrated that, in the prostate specific *Pten*-knockout mouse model, this type of deletion results in prostatic hyperplasia. In this model, a shift in the balance of differentiation was showed. Complete *Pten* inactivation is also observed in primary prostate tumors in human. This study showed that hyperplastic cells in *Pten*-knockout mice overexpress CK8, CK19, and Sca-1 which is characteristic for luminal epithelial progenitor cells. The obtained data led researchers to conclusion that luminal epithelial progenitor cells identified in the study are strong candidates for tumor initiating cells in the *Pten*-knockout prostate cancer model [84].

Moreover, Wang *et al.* [85] demonstrated that in the castrated mouse a rare luminal cell persists which expresses Nkx3.1 (regulator of prostate epithelial differentiation, marker of stem cell population). These cells are defined as CARNs (castration-resistant Nkx3.1-expressing cells). The lineage-tracing assay was used to identify CARNs as rare luminal epithelial population, possessing stem cell properties in prostate regeneration. CARNs cells were demonstrated to self-renew, and reconstitute prostate ducts in renal grafts after transplantation in immune-deficient mice. Eventually, a deletion of the *Pten* gene in CARNs resulted in rapid formation of carcinoma. These observations might indicate a novel luminal stem cell population as a cell-of-origin in prostate cancer [85].

Another group has recently performed a lineage-marking of basal and luminal cells to determine whether these cells contribute to tumors in a diverse range of mouse models. Their study showed that luminal cells are favored as the cell of origin for prostate cancer, however, explanted basal cells from these mice can generate tumors in grafts, after differentiation into luminal cells [86]. This results lead to the conclusion that both luminal and basal prostate cells can be a potential cellular origin for prostate cancer.

The hierarchy theory of the origin of prostate cancer suggests that rare tumor initiating cells can be identified in the population of whole tumor mass and they are distinct from the other cells. This model also predicts that the eradication of the stem cells will lead to complete eradication of the tumor [3,76,77,87,88,89].

The identification of these cells depends on the understanding of prostate cell differentiation lineage during development as well as adult prostate epithelium renewal. On the basis of this knowledge, the isolation and characterization of prostate cancer stem cells will become a true possibility and it should provide an explanation for the known clinical and molecular heterogeneity of human prostate cancer.

### 4.3. The Prostate Cancer Stem Cell Niche

The cancer stem cell hypothesis can partially explain the minimal residual disease occurrence. After radical prostatectomy, the persistence of one single cell in an appropriate environment could be the source of relapse. Several studies suggest the existence of functional microenvironments that support CSCs which is called the CSC niche. The signals that originate from this tumor niche regulate CSCs self-renewal, survival, and ability to invade tissues and the metastases development [18,20,74,90,91].

Microenvironment of the tumor might play the critical role in maintenance of stem-like features of prostate cancer cells. Cancer cell subpopulations can interact with other normal cells which are present in the tumor environment cooperating with them for benefits or even more often, taking advantage of them. Mateo *et al.* [92] recently described the co-operative interactions between the two clonal subpopulations of the PC-3 prostate cancer cell line. They found out that the invasiveness of a cancer stem cell enriched subpopulation is enhanced by a non-CSC subpopulation and can result in a significant increase of tumorigenic and metastatic potential of cancer stem cells. The knockdown and complementation experiments supported that SPARC protein (osteonectin, a matricellular glycoprotein, regulating tissue repair, and remodeling the extracellular matrix) is the main factor mediating the cooperation between CSCs and non-CSCs [92].

Furthermore, it has been observed that some changes in the tumor environment such as hypoxia, may induce, through HIF (hypoxia inducing factor) generation, the reprogramming of prostate cancer cells and increase the expression of stemness markers like CD44, Oct-3/4, Nanog, and drug resistance-associated molecules or anti-apoptotic proteins [93,94,95,96].

Eventually, CSC niches can also exist or be created in other, even distant from the primary tumor growth, locations. It is believed that a subpopulation of circulating tumor cells exists, which can settle down in the new environment such as bone marrow. The cells which create this microenvironment are defined as disseminated tumor cells (DTCs) [97].

Some patients that appear cured of prostate cancer can develop bone metastases even years after radical prostatectomy. This phenomenon indicates that prostate cancer cells can metastasize very early, and are present in the bone marrow at the time of surgical resection. For many years, these cells remain dormant, until they develop clinically detectable metastases. DTCs are a heterogeneous population but some of them might have characteristics of cancer stem cells. It has been revealed that the microenvironment within the bone marrow plays a crucial role in the process of metastasis [29,30,31,98]. It has been recently discovered that DTCs take over the bone marrow hematopoietic stem cells niche, and even direct competition for the niche was demonstrated. Eventually, it was shown that disseminated tumor cells affect the function of bone marrow, while settled in the marrow niche [30].

Some cells in the tumor microenvironment such as macrophages, endothelial cells, and fibroblasts may support prostate cancer progression. It was demonstrated that the bone marrow-derived mesenchymal stem cells (BM-MSCs) also participate in this process. Luo *et al.* [99] found out that BM-MSCs could be recruited into prostate tumor and lead to the increase of metastatic ability by the augmentation of cancer stem cell population respectively. Prostate cancer cells when cocultured with BM-MSCs grew as floating spheres which is characteristic feature of stem cells. They also showed that BM-MSCs coculture led to increased expression of stem cell markers such as CD133, OCT4, and Sox2 in the prostate cancer cell population [99].

The results of these findings confirm the importance of the tumor microenvironment and cancer stem cell niche during carcinogenesis and metastasis formation.

### 4.4. Markers of Prostate Cancer Stem Cells

For the distinction of prostate cancer stem cells from the other cells in tumor, several candidate markers have been tested. The most important are CD24, CD44 [69], CD49f [59], CD133 [35,61], CD166 [71], and α_2_β_1_ integrins [35]. Those markers have been tested alone and in several combinations but the ideal combination has not been found yet, which could clearly lead in the all cases to distinct cancer stem cells. The cause of this situation is the considerable diversity in the tumor histotypes and their genetic heterogeneity [35,71,100].

CD133 (Prominin-1 or AC133) is a cell surface glycoprotein whose biological function is poorly characterized, except that it interacts with cholesterol in cell membrane and is a marker of cholesterol-based “lipid raft” [101]. This membrane protein has been identified first as hematopoietic stem cell marker [102]. Richardson *et al.* [61] showed that approximately 1% of normal human prostate basal cells express the marker CD133, and these cells when restricted also to α_2_β_1_^hi^ population, possessed high *in vitro* proliferative potential. Moreover, this group demonstrated the ability of α_2_β_1_^hi^/CD133 normal prostate stem cells to form acini with prostatic—specific differentiation when grafted into athymic, nude mice [61]. CD133 has been proposed to be a putative surface marker in a number of tumors, however Collins *et al.* [35] used this marker to identify the prostate cancer stem cell population, and reported for the first time the identification and characterization of a cancer stem cell population from human prostate tumors. Cancer stem cells isolated by this group had a CD44^+^/α_2_β_1_^hi^/CD133^+^ phenotype, and possessed a significant capacity for self-renewal [35]. CD133 is often expressed in adult stem cells and it is believed that CD133—cholesterol microdomains might be implicated in the determination of cell fate and maintaining stem cell properties [103]. It has been investigated, using a series of anti-CD133 monoclonal antibodies, that attachment and growth of normal CD133^+^ prostate epithelial cell cultures requires expression of full-length glycosylated CD133 protein. In contrast to normal adult stem cells, prostate cancer stem cells do not require functional CD133 [103,104].

CD133^+^ prostate cancer stem cells have been also identified based on their integrin expression pattern. Rentala *et al.* [105] have recently identified the role of integrin profile in the prostate cancer stem cells isolated from the tissue specimens of patients with prostate cancer. They showed that the levels of β_1_ and α_2_β_1_ integrins were significantly higher than those of other integrins. CD133^+^ cells presented an increased degree of attachment to extracellular matrix protein. The study revealed for the first time the importance of the role of α_1_ and β_1_ integrins in the homing and differentiation of prostate cancer stem cells *in vitro* [105].

CD44 is a glycoprotein which exists as a standard isoform and a range of variant isoforms that are produced as a result of extensive alternative splicing. This alternative splicing mechanism and expression of variant isoforms contributes to uncontrolled tumor cell proliferation and transformation and induces metastasis formation [90,106,107]. CD44 antigen was first described as a lymphocyte homing receptor but it can be expressed by a wide range of cells. It belongs to the family of cartilage link proteins, while the ligands for this protein are collagen, laminin, fibronectin, E-selectin, L-selectin, and the extracellular matrix glycosaminoglycan hyaluronic acid (HA) [90,108,109,110]. In one of the studies performed *in vitro* and *in vivo* on cell lines and xenograft tumor models, Patrawala *et al.* [70] showed that CD44^+^ prostate cancer stem cells have stem—like properties such as increased clonoge nic and metastatic potential. These cells can form colonies in soft agar and have the potential to form tumors in NOD/SCID mice. It was concluded that this population of CD44^+^ stem cells is a heterogeneous population where primitive cells coexist with later progenitor cells [70]. These results provided convincing evidence that CD44 is associated with stem cells in prostate tumors.

In 2008, Hurt *et al.* [69] defined human prostate CD44^+^CD24^−^ subpopulation as prostate cancer stem cells with the ability to grow as nonadherent spheres in serum replacement medium. Only CD44^+^CD24^−^ population, but not CD44^+^CD24^−^ depleted population, had the potential to form tumors in NOD/SCID mice. This study defined an additional marker for prostate cancer stem cells and identified an almost homogenous population of stem cells with preserved colony—initiation ability [69]. Recent papers identified CD44^+^CD24^−^ cells in different prostate cancer cell lines [111,112]. These reports showed that both in primary and established prostate cancer cell lines, cancer stem cells can be more invasive. The data revealed that CD44^+^ subpopulation of stem-like cells actively invaded Matrigel, while CD44^−^ cells were characterized by a lack of invasiveness. Moreover, CD44^+^ cells were more tumorigenic when transplanted in NOD/SCID mice compared with non-invasive CD44^−^ cells [111].

Jiao *et al.* [71] identified subsequently CD166 as a surface molecule that can enrich sphere forming activity of Lin^−^Sca1^+^CD49f^hi^ population of cells in murine model. The aforementioned phenotype cannot be used for isolation of human cancer stem cells, because Sca-1 is only expressed in the mouse. However, CD166 is expressed in human organs and can be upregulated in certain tumor types. Therefore, this group found out that CD166 expression is upregulated in human prostate cancer, particularly in castration-resistant prostate cancer subpopulations. Similar to previous findings with murine prostate cells, the sphere forming activity was identified in the cells expressing high levels of CD166 surface molecule [71].

Altogether, all these studies of cell markers suggest that cancer stem cells may be derived from normal stem cells, since the expression patterns in these two cell types are very similar.

## 5. Implications for Prostate Cancer Treatment

Prostate cancer is one of the most common malignancies in male patients. Despite the progress that has been made in understanding of the molecular basis of carcinogenesis, and the introduction of early diagnostics protocols or effective therapeutic intervention, the disease progression to invasive and metastatic castration-resistant prostate cancers (mCRPCs) is still observed. Moreover, it is almost always associated with poor prognosis. In the beginning, advanced prostate cancer is treated with androgen deprivation therapy (ADT). Although it is initially very effective, it will finally lead to the development of aggressive and usually incurable conditions [1,2,113,114]. The current anti-androgen therapy and chemotherapy against CRPCs show limited survival benefits, because these types of treatment target primarily the bulk of neoplastic, fast-growing cancer cells but not cancer stem cells [115,116].

Prostate cancer stem cells are resistant to hormonotherapy, chemotherapy, and radiotherapy, so cancer relapse may be due to preferential killing of more differentiated cells while leaving undifferentiated stem cancer cells. Currently existing therapies often lead to an increase of resistant cancer stem cell subpopulation by selecting the most resistant clones within a heterogeneous population of cancer cells [116,117,118].

Several mechanisms for the development of CRPC have been described, most of which are based on the AR signaling regulation. Therefore, targeting the dysregulation of AR signaling in prostate cancer cells has been among the main interests in prostate cancer research [119,120,121,122,123].

Kregel *et al.* [124] have recently investigated the role of Sox2 (Sex determining region Y-box 2) transcription factor in normal and malignant prostate cancer cells. Sox2 is an oncogene and the fundamental regulator of the survival and pluripotency of stem cells, promoting more aggressive tumor phenotypes. Prostate tumors which are Sox2 positive have also high score in Gleason scale. They revealed that the expression of Sox2 was repressed by AR signaling in castration-resistant prostate cancer cells lines. However, AR—mediated repression of Sox2 expression can be reversed by the treatment with the anti-androgen factor. Moreover, in the castration-sensitive cell line which does not express Sox2 and does not normally form tumors in castrated nude mouse, lentiviral Sox2 expression was sufficient to significantly increase tumor formation in a castrated host [124]. This model points to the eventuality that the increased castration-resistant tumor formation may be due to the promotion of cancer stem cell proliferation and survival by Sox2 increased expression.

Past findings beg the question why the cells can survive androgen deprivation therapy which eventually leads to tumor relapse? Recent studies have revealed that AR splice variants without ligand-binding domain, can be found in androgen independent cell lines. These other forms of the receptor are usually constitutively expressed, therefore their activity is not regulated by androgens [125,126,127,128]. Tumor relapse and metastatic potential was shown to be connected to EMT phenotype (epithelial-to-mesenchymal transition). Moreover, EMT phenotype is linked with androgen deprivation therapy application [129]. Kong *et al.* [130] showed that overexpression of one of the AR variants—AR3 led to induction of EMT phenotype and is also involved in the regulation of the expression of stem cells marker genes. Besides, it was showed that androgen deprivation therapy enhanced AR and AR spliced variants expression leading authors to the conclusion that this upregulation is involved in progression to castration-resistant prostate cancer [130].

There is still a pressing need for the discovery of more effective therapy for advanced prostate cancer which would target the CSCs. Interestingly, metformin, a common oral biguanide used to treat type 2 diabetes, has been demonstrated to have anticancer effects as well. It was shown that metformin acts selectively on CSCs in several types of cancer among others: breast cancer, pancreatic cancer, colon cancer and finally prostate cancer. Metformin targets the mitochondria and reduces ATP production by oxidative phosphorylation which is the main energy source in CSCs [131,132,133,134,135]. Therefore metformin could be used to increase CSC sensitivity to existing therapies, enhance treatment efficacy and prevent relapses. Bilen *et al.* [136] has observed, in a case series, that metformin used alone or in combination with Zyflamend (herbal extract containing turmeric, holy basil, green tea, oregano, ginger, rosemary, Chinese goldthread, hu zhang, barberry, and basil skullcap) decreased level of PSA in prostate cancer patients in metastatic stage of disease. Authors proposed this treatment as a maintenance therapy for castration-resistant prostate cancer patients. Metformin and/or Zyflamend presumably target cancer stem cells and the tumor niche and keep the cancer in a dormant state [136].

Iliopoulos *et al.* [133] showed that metformin acts together with several chemotherapeutic agents to prevent relapse in xenografts generated with prostate and lung cancer cell lines. Consequently, it could be used as a part of combined therapy or to reduce the chemotherapy dose in patients treated for prostate cancer [133].

Furthermore, several articles have been published regarding phytochemicals and plant extracts which are able to target and selectively eliminate CSCs in many different types of cancer. It has been shown that sulforaphane, curcumin, piperine, β-carotene, and *Sasa quelpaertensis* extract exhibit not only anti-tumor properties but also are able to kill CSCs [137,138]. In relation to prostate cancer, anti-CSC effects were observed in the case of curcumin, the principal bright-yellow colored curcuminoid of turmeric (a plant of the ginger family). Curcumin was showed to cause effect on cell death and proliferation mediated by Wnt signaling in androgen-dependent and androgen-independent prostate cancer cell lines [139]. Recently Botchkina *et al.* [140] reported that structural analog of curcumin as a single agent or in combination with new-generation taxoid can exhibit significant activity against prostate CD133^hi^/CD44^+/hi^ cells. Moreover these drugs have been shown to inhibit expression of stem-related genes and even to induce expression of silent genes, which could potentially reverse drug resistance in these cells [140].

These recent findings support a better understanding of the prostate cancer stem cells’ role in the process of tumorigenesis. However, it is still essential to investigate the signaling pathways that regulate the self-renewal and survival of CSCs. In the future, such research may help to introduce novel therapeutic strategies for eliminating aggressive tumor cells and possibly prolong survival in patients with CRPC.

## 6. Conclusions

The stem cell theory provides a new framework for viewing the molecular mechanisms that underlie cancer cell hierarchy and heterogeneity within tumor mass. Clear understanding of the lineage hierarchy in prostate cancer cells will provide insights into the properties and characteristics of the origin of prostate cancer cells. An increasing body of evidence has shown that cancer stem cells represent a significant effort to effective cancer treatment in view of resistance to currently used clinical therapy. However, there is still a pressing need for the discovery of unique cancer stem cell markers to distinguish the cancer stem cells from the normal stem cells. But the ultimate challenge in the next few years will be developing new stem cell—directed drugs and reducing risk of relapse.
